# Supplementary figures and images for: Bioinformatic analysis of metastasis-associated metabolic landscape reveals an oncogenic role for the transsulfuration pathway
Source: Bioinform Adv. 2026 Mar 21;6(1):vbag084. doi: 10.1093/bioadv/vbag084 (PMC13037812; doi:10.1093/bioadv/vbag084)

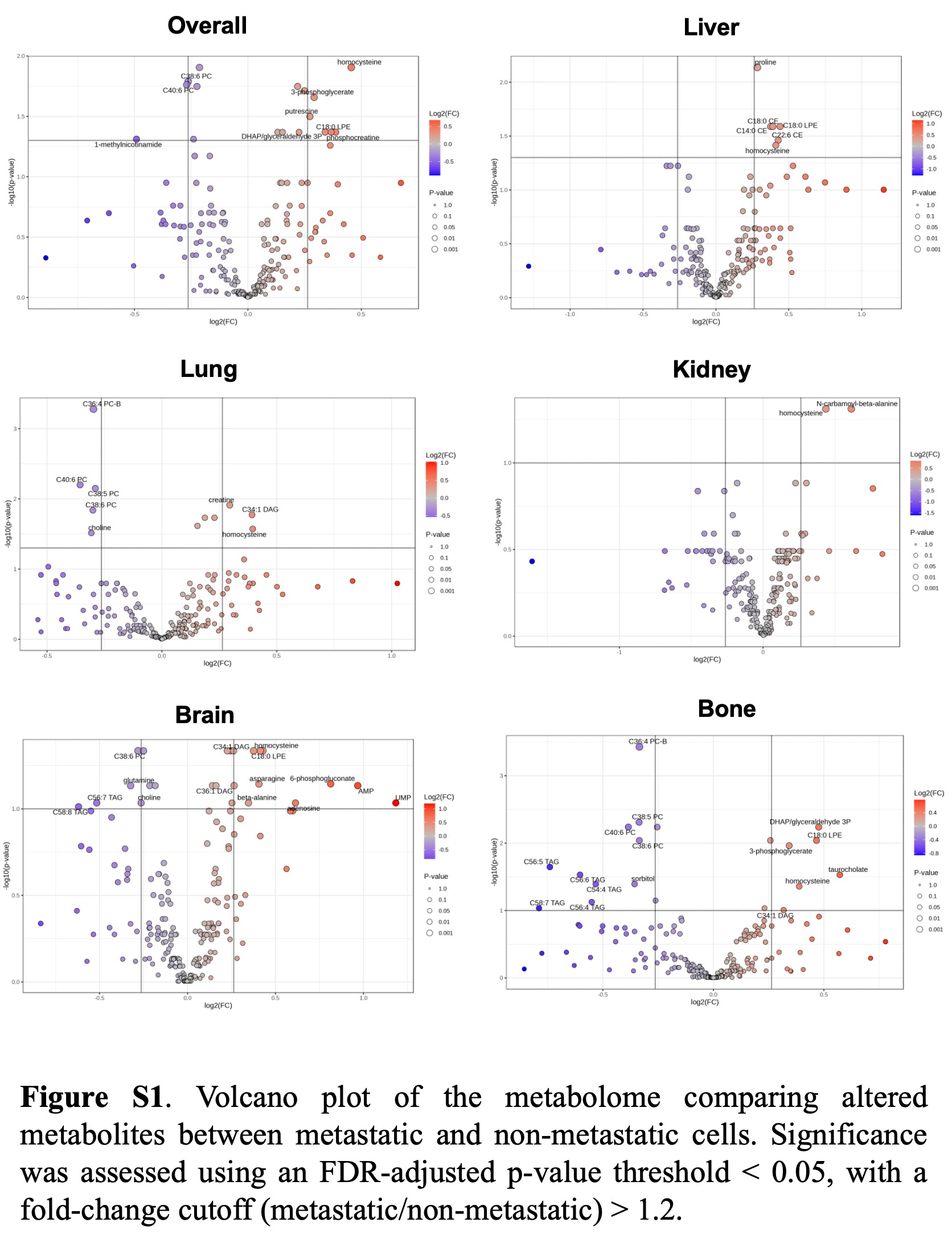

Supplement: vbag084_Supplementary_Data [file vbag084_supplementary_data.zip › FigS1.jpg]

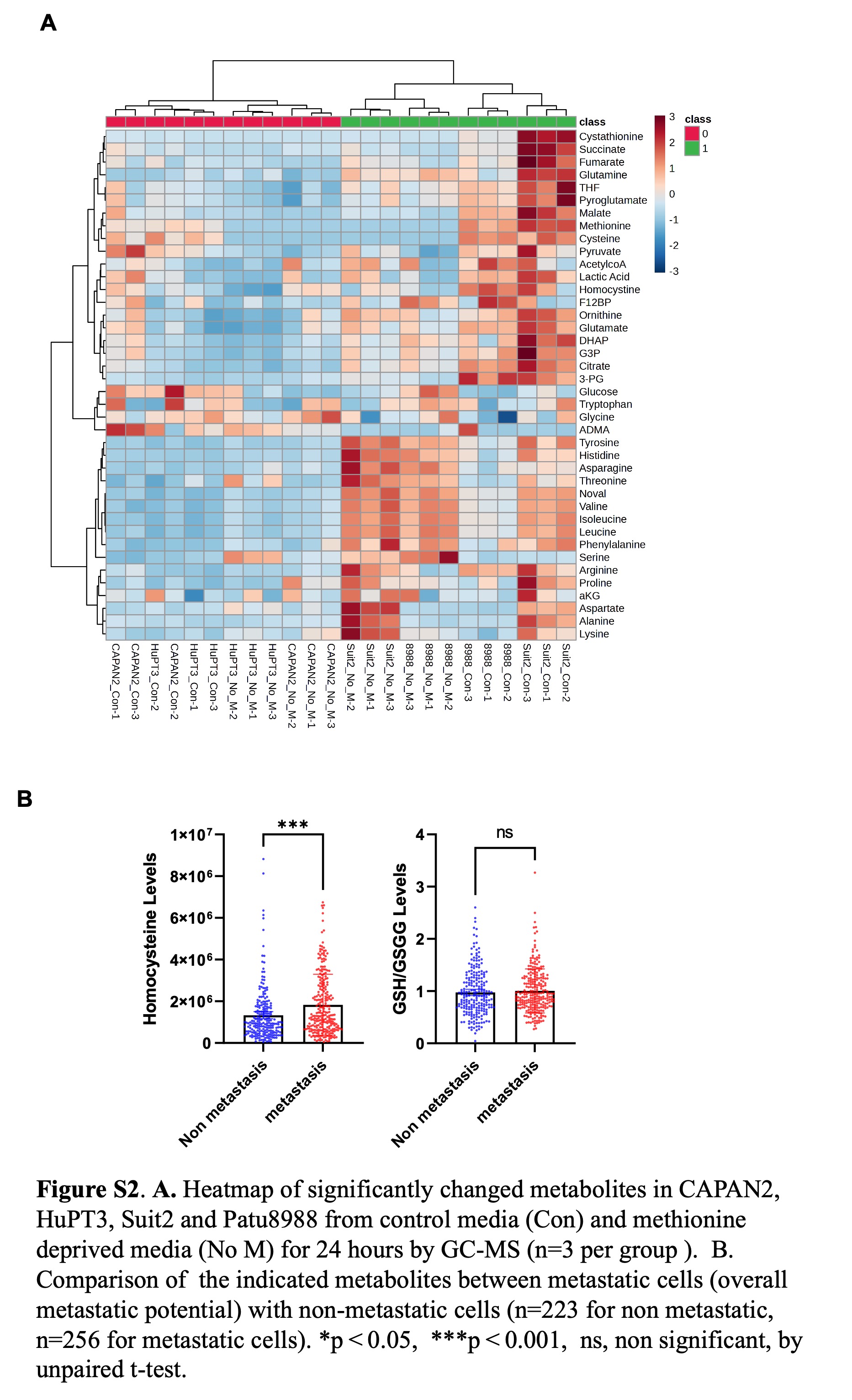

Supplement: vbag084_Supplementary_Data [file vbag084_supplementary_data.zip › FigS2.jpg]

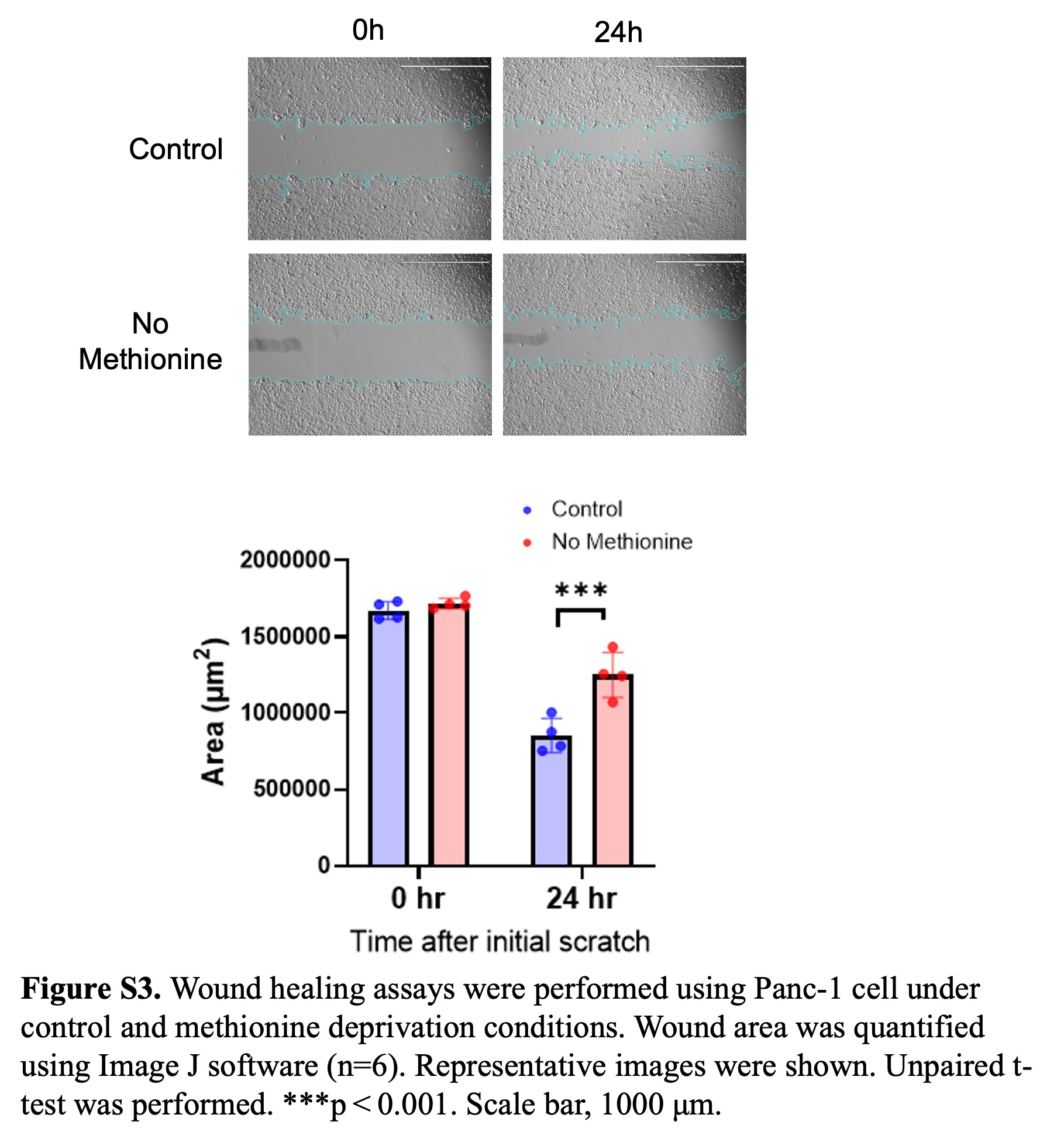

Supplement: vbag084_Supplementary_Data [file vbag084_supplementary_data.zip › FigS3.jpg]
